# Hybrid or Mixed Myelodysplastic/Myeloproliferative Disorders – Epidemiological Features and Overview

**DOI:** 10.3389/fonc.2021.778741

**Published:** 2021-11-16

**Authors:** Andrea Kuendgen, Annika Kasprzak, Ulrich Germing

**Affiliations:** Department of Hematology, Oncology, and Clinical Immunology, Heinrich-Heine-University Hospital Duesseldorf, Duesseldorf, Germany

**Keywords:** MDS/MPN, overlap syndromes, CMML, MDS/MPN-RS-T, aCML, JMML, MDS/MPN-U, epidemiology

## Abstract

The WHO-category Myelodysplastic/Myeloproliferative neoplasms (MDS/MPNs) recognizes a unique group of clonal myeloid malignancies exhibiting overlapping features of myelodysplastic as well as myeloproliferative neoplasms. The group consists of chronic myelomonocytic leukemia (CMML), atypical chronic myeloid leukemia, *BCR-ABL1*-negative (aCML), juvenile myelomonocytic leukemia (JMML), myelodysplastic/myeloproliferative neoplasm with ringed sideroblasts and thrombocytosis (MDS/MPN-RS-T), and myelodysplastic/myeloproliferative neoplasms, unclassifiable (MDS/MPN-U). The most frequent entity in this category is CMML, while all other diseases are extremely rare. Thus, only very limited data on the epidemiology of these subgroups exists. An appropriate diagnosis and classification can be challenging since the diagnosis is still largely based on morphologic criteria and myelodysplastic as well as myeloproliferative features can be found in various occurrences. The diseases in this category share several features that are common in this specific WHO-category, but also exhibit specific traits for each disease. This review summarizes published data on epidemiological features and offers a brief overview of the main diagnostic criteria and clinical characteristics of the five MDS/MPN subgroups.

## Introduction

The World Health Organization (WHO) recognizes a group of rare clonal hematopoietic malignancies with mixed features of Myelodysplastic Syndrome (MDS) as well as Myeloproliferative Neoplasms (MPNs) (1). These malignancies are placed in a separate WHO-category named myelodysplastic/myeloproliferative neoplasms (MDS/MPN). The group consists of myeloid diseases including chronic myelomonocytic leukemia (CMML), atypical chronic myeloid leukemia, *BCR-ABL1*-negative (aCML), juvenile myelomonocytic leukemia (JMML), myelodysplastic/myeloproliferative neoplasm with ringed sideroblasts and thrombocytosis (MDS/MPN-RS-T), and myelodysplastic/myeloproliferative neoplasms, unclassifiable (MDS/MPN-U). Diagnosing these diseases can be challenging, as they can exhibit different features of MDS and MPNs. While the simultaneous existence of dysplasia and proliferation is mandatory, other features might be cytopenias, often in coexistence with “cytoses” and organomegaly. “MDS-like” symptoms as a result of ineffective hematopoiesis including fatigue, dyspnea, infections, and bleeding occur in parallel to the more “MPN-like” symptoms resulting from proliferative hematopoiesis, namely night sweats, weight loss, and increased risk of thromboembolic complications. Unfortunately, the morphological features of MDS/MPNs are not specific but can be found in other myeloid malignancies at presentation or as part of disease progression. Diagnostically, there is a considerable overlap between the different MDS/MPNs as well as the different myelodysplastic and myeloproliferative neoplasms. At present, no cytogenetic or molecular genetic abnormalities specific for any of the MDS/MPN subtypes exist. Nevertheless, genetic abnormalities play an important role in excluding a diagnosis of a particular MDS/MPN and some abnormalities might at least help ascertain the correct subtype (2–17).

The existence of disorders with overlapping myelodysplastic and myeloproliferative features has been described years ago. A true recognition and classification of this group of diseases, however, occurred much more recently. In 1976 the French-American-British (FAB) cooperative group introduced a classification and nomenclature of the acute myeloid and lymphoid leukemias (18). Two types of MDS, RAEB and CMML, were presented as an addendum. Then, in 1982, the FAB-group introduced a classification and nomenclature of the MDS (19). CMML was included as one of the 5 subtypes of MDS in this classification system. Only with the introduction of the WHO-classification (20) in 2001 the existence of overlap syndromes between MDS and MPN was formally recognized and CMML was moved into this new founded category of myeloid malignancies. In addition to CMML the new group included aCML, JMML, and MDS/MPN-U. Refractory anemia with ringed sideroblasts associated with marked thrombocytosis (RARS-T) was initially proposed as a provisional entity in the WHO 2001 classification of myeloid neoplasms (20) and only 2016 recognized as a formal subgroup (MDS/MPN-RS-T) of MDS/MPN by the latest version of the WHO-classification (1). Additional entities that have been discussed and might represent separate entities of MDS/MPN in future classifications are MDS with isolated del(5q) and *JAK2-V617F* mutation and MDS/MPN with isolated isochromosome 17q (21–30).

The WHO-category MDS/MPN encompasses three relatively well-defined entities, namely CMML, JMML, and MDS/MPN-RS-T, the diagnostic criteria for which are easy to follow. In contrast, aCML and MDS/MPN-U are less well-defined and their diagnosis largely remains a matter of exclusion of other myeloid neoplasms (1). Main diagnostic criteria of the MDS/MPN subgroups according to WHO 2016 are depicted in **Table 1**.

**Table 1:** Main diagnostic criteria of the MDS/MPN subgroups according to WHO 2016 (1).

CMML	JMML	MDS/MPN-RS-T	aCML	MDS/MPN-U
Persistent peripheral monocytosis (≥1000/µl) with monocytes accounting for ≥10% of leukocytes (1)	Peripheral blood monocyte count (≥1000/µl) (required)	Anemia associated with erythroid lineage dysplasia, with or without multilineage dysplasia; ≥15% ringed sideroblasts	Peripheral blood leukocytosis ≥13000/µl, due to increased numbers of neutrophils and their precursors (i.e., promyelocytes, myelocytes, and metamyelocytes), with neutrophil precursors constituting ≥10% of the leukocytes	Myeloid neoplasm with mixed myeloproliferative and myelodysplastic features at onset, not meeting the WHO criteria for any other myelodysplastic/myeloproliferative neoplasm
- Dysplasia involving ≥ myeloid lineage or - if myelodysplasia is absent or minimal, criteria (1-4) are met and - an acquired, clonal cytogenetic or molecular genetic abnormality is present in hematopoietic cells or - the monocytosis has persisted for ≥3 months and all other causes of monocytosis (i.e., malignance, infection, and inflammation) have been excluded	Splenomegaly (required)	Persistent thrombocytosis, with platelet count ≥450000/µl	- Dysgranulopoiesis, which may include abnormal chromatin clumping - No or minimal absolute basophilia; basophils constitute <2% of the peripheral blood leukocytes - No or minimal absolute monocytosis; monocytes constitute <10% of the peripheral blood leukocytes - Hypercellular bone marrow with granulocytic proliferation and granulocytic dysplasia, with or without dysplasia in the erythroid or megakaryocytic lineages	- Clinical and morphologic features of one of the myelodysplastic syndromes - Clinical and morphologic myeloproliferative features manifesting as platelet count of ≥450000/µl associated with bone marrow megakaryocyte proliferation and/or a white blood count of 13000/µl
- WHO-criteria for *BCR-ABL1*-positive chronic myeloid leukemia, primary myelofibrosis, polycythemia vera, and essential thrombocythemia are not met (2)	- No Philadelphia chromosome or *BCR-ABL1* fusion (required)	- No *BCR-ABL1* fusion - No history of myeloproliferative neoplasm, myelodysplastic syndrome (except myelodysplastic syndrome with ringed sideroblasts), or other myelodysplastic/myeloproliferative neoplasm	- WHO-criteria for *BCR-ABL1*-positive chronic myeloid leukemia, primary myelofibrosis, polycythemia vera, and essential thrombocythemia are not met	- WHO-criteria for *BCR-ABL1*-positive chronic myeloid leukemia, primary myelofibrosis, polycythemia vera, and essential thrombocythemia are not met - No history of recent cytotoxic or growth factor therapy that could explain the myelodysplastic/myeloproliferative features
- No rearrangement of *PDGFRA, PDGFRB, PCM1-JAK2*, and *FGFR1* (must be specifically excluded in cases of eosinophilia) (3)	- Somatic mutation in *PTPN11, KRAS*, or *NRAS* - Clinical diagnosis of neurofibromatosis type 1 or *NF1* mutation - Germline *CBL* mutation and loss of heterozygosity of *CBL* (1 genetic criterion is sufficient) Cases that do not meet any of the genetic criteria must meet the following criteria in addition to the clinical and hematological criteria: - Monosomy 7 or any other chromosomal abnormality or ≥2 of the following: - Increased hemoglobin F for age - Myeloid or erythroid precursors on peripheral blood smear - Granulocyte-macrophage colony-stimulating factor (CSF2) hypersensitivity in colony assay - Hyperphosphorylation of STAT5	- SF3B1 mutation or, in the absence of SF3B1 mutation, no history of recent cytotoxic or growth factor therapy that could explain the myelodysplastic/myelo-proliferative features - no rearrangement of *PDGFRA, PDGFRB, PCM1-JAK2*, and *FGFR1* (must be specifically excluded in cases of eosinophilia) and - no t(3;3)(q21.3;q26.2), inv(3)(q21.3;q26.2), or del(5q)	- No rearrangement of *PDGFRA, PDGFRB, PCM1-JAK2*, and *FGFR1* (must be specifically excluded in cases of eosinophilia)	- No rearrangement of *PDGFRA, PDGFRB, PCM1-JAK2*, and *FGFR1* (must be specifically excluded in cases of eosinophilia) - No del(5q)
Blasts constitute <20% of the cells in the peripheral blood and bone marrow (4)	Blasts constitute <20% of the cells in the peripheral blood and bone marrow (required)	<1% blasts in the peripheral blood and <5% blasts in the bone marrow	Blasts constitute <20% of the cells in the peripheral blood and bone marrow	Blasts constitute <20% of the cells in the peripheral blood and bone marrow

The entities included within the WHO-category MDS/MPN share several common features, but also exhibit differences defining the individual disease (13, 15, 31–54). The hallmark of MDS/MPNs is the unique mixture of cytopenias and “cytoses”. Therefore, the bone marrow is typically hypercellular due to the combination of a very effective “myeloproliferative” hematopoiesis and an ineffective, dysplastic hematopoiesis (54–57). Dysplasia is seen in at least one hematopoietic lineage. By definition, the diseases have further characteristics in common: The percentage of blasts in PB and BM must be <20%. Certain cytogenetic abnormalities must be ruled out to exclude other genetically defined myeloid malignancies sharing features of myelodysplastic and myeloproliferative diseases. These include *BCR-ABL1, PDGFRA, PDGFRB, PCM1-JAK2*, and *FGFR1* (1). Except JMML, MDS/MPN are diseases of the elderly and all MDS/MPN show a clear male preponderance, with the possible exception of MDS/MPN-RS-T where the gender distribution differs between publications and some even exhibit a slight predominance of the female gender (13, 15, 31–53). A high frequency of anemia is a further characteristic of most MDS/MPN, while other cytopenias are often less pronounced when compared to MDS, or “cytoses” occur. An increased WBC is frequent or mandatory in proliferative CMML, JMML, aCML, MDS/MPN-U and MDS/MPN-RS-T. Thrombocytosis is mandatory in MDS/MPN-RS-T and can occur in all other MDS/MPN subtypes as well (1, 50–54, 57).

The majority of patients show fatigue and most, maybe except MDS/MPN-RS-T, exhibit frequent general (MPN-like) symptoms like night sweats as well as symptoms of organomegaly and extramedullary disease. Spleno- and often an additional hepatomegaly are frequent clinical findings, especially in CMML and JMML. Again, an exception might be MDS/MPN-RS-T, but the data on these clinical features is unfortunately sparse regarding this rare entity (15, 17, 41, 43, 44, 46, 48–54, 58).

MDS/MPN also share a very low frequency of cytogenetic aberrations compared to MDS. In this regard the exception might be MDS/MPN-U. If cytogenetic abnormalities occur, +8 is by far the most frequent (13, 14, 17, 37, 39–44, 46, 48–54, 58). The frequency of molecular abnormalities on the other hand is very high. Such aberrations can be found in more than 90% of patients (2–17, 50–54, 58). Except in MDS/MPN-RS-T and partly in MDS/MPN-U, the frequency of JAK-2 mutations is very low when compared to classical MPNs (50–54).

Unfortunately, another feature, shared by this group of overlap diseases, is a poor response to treatment other than allogeneic stem cell transplantation. CMML, aCML, and MDS/MPN-U share a poor prognosis in general and afflicted patients are often too old for transplantation (16, 17, 31–36, 41, 43, 44, 46–54, 59–61). JMML might have a special role, as some children show spontaneous regression and otherwise most afflicted patients can be transplanted, but on the other hand the severity of the disease is obvious regarding the still unsatisfying long-term survival and the poor response to treatment other than transplantation can be observed for JMML as well (32, 37, 62). The only true exception seems to be MDS/MPN-RS-T which exhibits a low risk of progression and a long median survival time (13, 39–42). An overview of differences and similarities between the MDS/MPN subgroups is given in **Table 2**.

**Table 2:** Differences and similarities between the MDS/MPNs.

	CMML	JMML	MDS/MPN-RS-T	aCML	MDS/MPN-U
Incidence	accounts for 12.5% of all MDS currently in the MDS registry (35) --------------------------------- ~0.3-0.4 (31, 33, 34, 62); 1 (36); 4.1 (32) cases per 100 000; --------------------------------- For comparison: incidence MDS 3-5/100 000 (34, 63–65)	--------------------------------- 0.1 per 100 000 (32), about 1 per million children per year (66); <2-3% of all leukemias in children, ~20-30% of all cases of MDS or MPD in children, 0.63 per million children per year (38) --------------------------------- For comparison: Incidence of leukemia in children ~6 per 100 000 (67) Incidence of childhood MDS 0.5-6/million population per year (37, 66, 68)	accounts for 1,45% of all MDS currently in the Duesseldorf MDS registry (35) --------------------------------- <1% of MDS (49); 0.7% of all MDS cases (50, 69); --------------------------------- For comparison: incidence MDS 3-5/100 000 (34, 63–65)	--------------------------------- 0.1 per 100 000 (32); 1-2% of bcr-abl positive CML (44, 70, 71) --------------------------------- For comparison: incidence bcr-abl+ CML ~0.7-1.5 per 100 000 (72, 73)	accounts for 0,05% of all MDS currently in the Duesseldorf MDS registry (35) --------------------------------- <2% of MDS (49); --------------------------------- For comparison: incidence MDS 3-5/100 000 (34, 63–65)
Median Age/years	75-76 (31–33)	<1 -2 (32, 37, 38)	63-75 (13, 39–42)	62-73 (15, 32, 43–47)	65-71 (16, 17, 41, 43, 48, 49)
Gender	61-76% male (31– 36)	71-73% male (32, 38)	47-62% male (13, 39–42)	55-69% male (32, 43, 46, 47)	64-76% male (16, 17, 41, 43, 48)
Bone marrow findings	current data from the Duesseldorf registry: cellularity: 68% hypercellular 27% normocellular 5% hypocellular fibrosis: 16% (35) --------------------------------- Degree of marrow fibrosis: 54% (74); ≥MF2 3% (75), ≥MF2 77% (76); possible presence of plasmacytoid dendritic cells (58, 77–79); Cellularity: hypocellular 5%, normocellular 30%, hypercellular 65% (50); differentiation between CMML 0, I, and II depending on marrow blast count	--------------------------------- Hypercellular marrow, often reduced megakaryocytes, normal- moderately elevated blast count (51)	current data from the Duesseldorf registry: cellularity: 67% hypercellular 31% normocellular 2% hypocellular fibrosis: 8% (35) --------------------------------- <5% marrow blasts [median 1-2% (13, 41, 42)], presence of >15% ringed sideroblasts, presence of large, atypical megakaryocytes, usually hypercellular marrow (13, 42)	--------------------------------- Degree of marrow fibrosis: 37% (15), 71% (52), 31% (43); median blast count 1-3% (15, 43–46); hypercellular marrow, myeloid hyperplasia, granulocytic dysplasia prominent, erythroid hypoplasia (52, 57)	current data from the Duesseldorf registry: cellularity: 50% hypercellular 50% normocellular 0% hypocellular fibrosis: 42% (35) --------------------------------- Degree of marrow fibrosis: 27% (43); Median marrow blast count 2-3% (16, 17, 41, 43, 48)
Peripheral cell counts	Absolute and relative monocytosis, pB blast count <20%, cytopenias may occur in all cell lines, differentiation between dysplastic and proliferative CMML by WBC (</>13000/µl); frequency dysplastic:proliferative 42%:58% (36), 50%:50% (50)	leukocytosis, monocytosis, immature monocytes, myelocytes, metamyelocytes, nucleated red cells, presence of peripheral blasts frequent (median <2%), often thrombocytopenia and moderate anemia (51)	WBC can be slightly decreased, normal or more often mild to moderately increased, thrombocytosis by definition, moderate to severe anemia (13, 39–42)	WBC increased, mostly mild to moderate anemia, absolute monocytosis is common, but the percentage of monocytes is low, no prominent basophilia (57)	WBC can be everything between decreased and strongly increased; platelet counts, and hemoglobin values can vary as well (16, 17, 43, 48)
Localization	High frequency of hepato- and splenomegaly, and extramedullary involvement (especially proliferative subtype) --------------------------------- 27% splenomegaly in the Duesseldorf MDS registry (35)	Almost 100% hepato-splenomegaly, frequently lymphadenopathy, skin rashes, and other extramedullary involvement	Frequency of hepato-, splenomegaly 12% (41) --------------------------------- 18% splenomegaly in the Duesseldorf MDS registry (35)	Frequency of splenomegaly 50-70% (15, 44, 46), frequency of 49% hepatomegaly (44)	Frequency of splenomegaly 23-36% (17, 41, 43, 48), Hepatomegaly 10-23% (41, 43) --------------------------------- 62% splenomegaly in the Duesseldorf MDS registry (35)
Clinical features	Frequently fatigue, night sweats, symptoms from organomegaly, bone pain, weight loss, cachexia (58), about 30% can present with autoimmune diseases/systemic inflammatory syndromes (80–83); hyperleukocytosis/leukostasis can occur	Increased synthesis of hemoglobin F; signs of autoimmunity in 25% of children, hypergammaglobulinemia in about 50% (37); often pulmonary infiltrates with dry cough, tachypnea, and interstitial infiltrates; frequent gut infiltrates with diarrhea and gastrointestinal infections, skin rashes, eventually features of syndromic disease like café-au-lait spots, facial dysmorphia, heart disease, failure to thrive, hearing loss, and others (51)	Frequently presenting with fatigue, increased risk of thromboembolic events, comparable to ET; “MPD-typical” general symptoms appear to be less frequent	Frequently fatigue, night sweats, symptoms from organomegaly, bone pain, weight loss, cachexia; hyperleukocytosis/leukostasis can occur	Constitutional symptoms 69% (48); 61% symptomatic (46% fatigue, 15% night sweats) (49)
Cytogenetic features	20-30% abnormal, most frequent +8, -7, del(7q), -Y (58)	About 35% abnormal, about 25% -7 (37)	About 10-20% abnormal, most common +8 (13, 14, 39–42)	20-44% abnormal, most frequent +8, del(20q) (43, 44, 46)	35-51% abnormal, most frequent +8, -7/del(7q) del(20q), and complex (17, 43, 48, 49)
Molecular genetic features	Frequency of molecular abnormalities >90% most frequent TET2 (~60%), SRSF2 (~50%), ASXL1 (~40%), RUNX1 (~15%), SETBP1 (~15%), CBL (~15%), NRAS (~15%), KRAS (~10%), SF3B1 (~5-10%), U2AF1 (~5-10%), IDH2 (~5-10%), ZRSR2 (~5%), PHF6 (~5%), PTPN11 (~5%), EZH2 (~5%), DNMT3A (~5%), FLT3 (~<5%), TP53 (~1%), IDH1 (~1%) (2–11, 58, 84); JAK-2 relatively uncommon	Mainly altered RAS pathway; about 90% of patients have mutations in either PTPN11, NRAS, KRAS, CBL, or NF1; secondary genetic alterations: ASXL1, EZH2, SETBP1, JAK3, spliceosomal genes (less frequent) (12)	SF3B1 (~85%), JAK2 (~50%), TET2 (~25%), ASXL1 (~20%), DNMT3A (~15%), SETBP1 (~10%), EZH2 (~7%), SRSF2 (~7%), U2AF1 (~5%), IDH2 (~4%), CBL (~4%), ZRSR2 (~3%), MPL (~3%), ETV6 (~3%), RUNX1 (~1%) (13, 14, 84)	SETBP1 (30%), ETNK1 (16%) ASXL1 (43%), TET2 (27%), NRAS/KRAS (22%), EZH2 (19%), RUNX1 (14%), SRSF2 (14%), CBL (8%), CREBBP (8%), CSFR3R (<5%) (15),; JAK-2 relatively uncommon (84)	ASXL1 (29%), TET2 (27%), JAK2 (25%), SRSF2 (23%), EZH2 (17%), U2AF1 (13%), RUNX1 (13%), SF3B1 (12%), ZRSR2 (11%), SETBP1 (11%), NRAS (10%), DNMT3A (9%), TP53 (8%) CSF3R (5%), STAG2 (5%), CBL (4%), ETV6 (4%), NPM1 (4%), CEBPA (4%), CALR (3%), KRAS (3%), PTPN11 (3%), MPL (3%) (16) ASXL1 (56%), SRSF2 (37%), SETBP1 (21%), JAK2 (19%), NRAS (15%), TET2 (13%) (17, 84)
survival	5-year survival 20% (31); 3-year survival 21% (34); 5-year survival 16% males ≥60, 27% males <60years old, 18% females ≥60, 32% females <60 years of age (32); RSR at 3-years 27-37%, 5-years 19-23% in the US and RSR at 3-years 48-40%, 5-years 34-26% in Switzerland (33); Median OS: not reached to 18 months (CPSS) (59), 97-16 months (Mayo Molecular Model) (60), and 56-9.2 months (GFM-Model) (61) --------------------------------- Current data from the Duesseldorf MDS registry (35): mOS: CMML 0: 33 months CMML I: 20 months CMML II: 14 months	5-year survival 56% males, 66% females (32); children with CBL-mutated JMML often experience spontaneous regression as well as a few patients with NRAS mutated JMML; the majority of patients requires allogeneic transplantation; allogeneic transplantation results in a disease-free survival of 52% (data from 2005) (62); survival at 10 years 0.39 for children after HSCT and 0.06 for children without HSCT (data from 1997) (37)	Median OS 76 months (39); median OS 80, 42, and 11 months in three different risk groups (13); median OS NR (41); median OS 88-101 month (42); median OS 10,7 years (40) --------------------------------- Current data from the Duesseldorf MDS registry (35): mOS: 61 months	5-year survival 11% males, 16% females ≥60 of age (32); median OS 25 months (44), Median OS 24 months, 5-year survival 7% (46); median OS 10 months, 2-year survival 28% (47); median OS 12 months (43)	Median OS 19 months (41), median OS 12 months (from sample date) (16); median OS 12.4 months (from presentation) (48); median OS 26 months (17); median OS 21 months (49); median OS 22 months (43)
Leukemic transformation	AML transformation rate 39% (36); 4-year leukemic transformation rate 0-48% (59); AML transformation 19% after a median of 7 months (46); AML transformation 8, 23, and 23% at 5 yrs (63); AML transformation 13, 29, 60, and 73% (59), AML transformation 16% at a median follow up of 23 months (60) --------------------------------- Current data from the Duesseldorf MDS registry (35): AML at 2 yrs: CMML O: 14% CMML I: 21% CMML II: 42% AML at 5 yrs: CMML O: 22% CMML I: 32% CMML II: 64%	No data available	1.8/100 patients/year (39, 40); 2% at a median follow-up of 27 months (13); AML transformation 7% (median time to AML or follow-up not given) (42) --------------------------------- Current data from the Duesseldorf MDS registry (35): AML at 2 yrs.: 5% AML at 5 yrs.: 8%	AML transformation 40%, median time to AML 18 months (44); AML transformation 31%, median time to AML 11.5 months (46); AML transformation 9%, median time to AML 5 months (47); AML transformation 37%, median leukemia free survival 11 months (43)	AML transformation 16% after a median of 11 months (16); AML transformation 16% after a median follow up of 61 months, median LFS 24 months (17); AML transformation 23%, median LFS 19 months (43); AML transformation 54% (70% of non-RARS-T MDS/MPN-U) after a median follow-up of 20 months (49)
Subtypes and closely related diseases	CMML associated with mastocytosis, CMML and blastic plasmacytoid dendritic cell neoplasm, CMML associated with eosinophilia (77–79), t-CMML (85), pre CMML syndromes (oligo-monocytic CMML) (86, 87), CMML with JAK-2 mutation, CMML with rearranged PDGFRA, PDGFRB, FGFR1, PCM1-JAK2, other MPN (MF, PV.) with monocytosis (“MPN with CMML-like phenotype”) (88)	Noonan Syndrome (51)		aCML with abnormal chromatin clumping (89–92)	MDS with del(5q) and *JAK2 V617F* mutation, MDS with isolated isochromosome (17q) (21–30)

## Epidemiology of MDS/MPN

Epidemiological studies on MDS/MPNs are scarce and most of the existing data is limited to CMML. For other MDS/MPN subtypes only vague estimations exist.

Regarding CMML, Dinmohamed et al. (31) find an annual standardized incidence (ASR) rate of 0.3 per 100.000 for the period of 1989 to 2012 in the Netherlands. The ASR increased from 1989 to 2007 and remained then stable at 0.38/100 000 until 2012. The ASR was higher in males (0.42) when compared to females (0.18) and increased with age from 0.02 per 100.000 under the age of 50 years to 3.62 in patients ≥80 years old. Relative survival did not improve over time with the 5-year relative survival rate (RSR) being only 16, 20, and 20% in the three time periods investigated. Interestingly, the RSR was poor in all age groups, ranging from 12% in the group above 80 years to 21% in patients younger than 50 years of age.

In a study on the epidemiology of MDS and MPDs in the United States from 2001 to 2004, data from the NAACCR as well as the SEER programs was used by Rollison et al. (34). They found an age adjusted incidence of 0.3 per 100 000, about one-tenth that of MDS. Survival of patients with CMML was worse when compared to MDS and MPD, the 3-year survival being 21%. While 3-year survival decreased with increasing age, similar to the Dutch study survival was again poor in all age groups. 3-year survival was 12% in patients over the age of 80 and, in comparison, still only 33% in patients with an age between 50 and 59 years. This was clearly inferior when compared to MDS (37% and 54%) and MPD (66 and 89%), respectively.

Another US study on Seer data reported incidence and survival of patients with MDS and MPDs between 2001 and 2012 (32). An age adjusted incidence rate of 4.3 per 100 000 was reported for all MDS/MPN taken together. The largest proportion represented by far the patients with CMML (4.1 per 100 000). Patients with aCML had an age adjusted incidence rate of 0.1 same as patients with JMML. Incidence rates for MDS/MPN-U or MDS/MPN-RS-T were not reported in this study. Incidence rates were higher in males when compared to females for all 3 overlap syndromes reported (male: female ratio 2.31 for CMML, 2.05 for aCML, and 2.30 for JMML). Incidence increased exponentially with increasing age. This was especially apparent for CMML when compared to MPNs. For aCML and JMML such data was not available. The incidence rates for CMML were significantly lower in Hispanics (3.0), Blacks (3.1), or Asian/Pacific Islanders (2.7) compared to Non-Hispanic Whites (4.4). For the other subgroups numbers were too low for such calculations. Incidence rates for CMML did not increase significantly over the time period investigated. As in the other studies survival of CMML patients was generally very poor, but slightly better in women *vs* men and younger *vs*. older patients (5-year survival 16% males ≥60, 27% males <60 years old, 18% females ≥60, 32% females <60 years of age). For aCML 5-year survival was even worse with 11% for male patients compared to 16% for female patients over the age of 60. In children with JMML the 5-year survival rate was 56% for males and 66% for females, without treatment data given.

In an investigation on the incidence of MDS in Western Greece during a 20-year period (1990-2009) Avgerinou et al. (36) found an incidence of MDS of 6 per 100.000 inhabitants. From the data given, a crude incidence of 1 per 100 000 can be calculated for CMML while the incidence is only 0.1 per 100 000 for all other MDS/MPD together. The incidence of CMML remained stable over the time period investigated. Within the period under investigation 39% of CMML patients progressed to AML.

In a comparative study between Switzerland and the US (SEER-data) Benzarti et al. described epidemiological trends regarding CMML between 1999 and 2014 (33). The age standardized incidence was similar and remained relatively stable in both countries, being 0.32 (1999-2006) and 0.38 (2007-2014) in Switzerland and 0.37 and 0.35 in the US. In both countries and time periods it was much higher in patients above the age of 75 (3.01-4.83 ≥75 *vs*. 0.17-0.25 <75 years of age) and higher in males when compared to females (0.51-0.57 *vs*. 0.17-0.25). There were an increasing proportion of older patients ≥75 years of age observed in the Swiss Cancer Registry compared to a decreasing in the US SEER database. Relative survival improved significantly in the US database (3-years 27-37%, 5-years 19-23%) and remained stable in Switzerland (3-years 48-40%, 5-years 34-26%).

In our MDS registry CMML accounts for about 12.5% of all MDS during a period from 1982 to 2020, leading to a rough incidence of 0.4 per 100.000 that remained relatively stable over the investigated time period (35).

CMML is by far the most frequent of MDS/MPNs. Published incidence rates range from 0.3-4.1 per 100 000 inhabitants with a median age above 70 years and a male predominance (31–36, 63). CMML might be described as even more heterogeneous when compared to MDS, with hematological characteristics ranging from solely dysplastic forms, presenting often cytopenic and resembling MDS with peripheral monocytosis to very proliferative forms, characterized by high white blood cell counts, but also by splenomegaly, extramedullary involvement, and strong general symptoms. Therefore, the initial distinction, as proposed by the FAB-classification (19), between dysplastic and proliferative CMML remains useful from a clinical point of view. Diagnosis is based on the presence of sustained (>3 months) peripheral blood monocytosis, along with bone marrow dysplasia. In the current WHO-classification CMML is subdivided into 3 different groups (CMML 0-II) according to blast count (1, 93). In 386 patients from our Duesseldorf registry Schuler et al. found a distribution of 26% CMML-0, 53% CMML-I, and 21% CMML-II (94).

Chromosomal abnormalities are less frequent in CMML when compared to MDS and have been described in about 10-40% of cases. On the other hand, more than 90% of CMML patients exhibit molecular mutations. These are relatively homogenous compared to other myeloid malignancies and mostly belong to a subset of 20 frequently mutated genes (58, 61, 66, 95). The clinical course of CMML patients is extremely variable, with wide differences in survival and leukemic transformation risk. Generally, survival is low around 20-35% at 5 years (35, 36, 58, 60, 93), even in lower age groups, but varies between the different prognostic risk groups. In several studies on CMML prognosis (CPSS, Mayo- Molecular Model, GFM- Model) (59–61) median survival ranged from 56 months to not reached in the best and 9-18 months in the worst prognostic group. The risk of leukemic transformation is around 15% over 3-5 years (59–61), but again varies considerably between subgroups (4-year leukemic transformation rate 0-48% [CPSS-paper) (59)].

JMML is a clonal hematopoietic stem cell disorder of childhood. It is extremely rare with an incidence rate of about 1 per 1 000 000 children under the age of 14 years (12, 37, 38, 96). Like CMML the disease is characterized by proliferation of the monocytic lineage. The age at diagnosis can vary between 1 month and early adolescence, but at least 50% of children are below 2 years old and only 5% are 5 years or older (37). Splenomegaly occurs in almost all cases, and hepatomegaly, lymphadenopathy as well as extramedullary involvement including skin, lung, and gastro-intestinal tract are common. While JMML shares a number of features with CMML, its pathobiology is unique. About a third of patients have cytogenetic abnormalities, about a quarter show monosomy 7. Molecular abnormalities occur in at least 90% of patients and usually involve the RAS pathway. About 90% of cases belong to one of 5 groups with mutations in either *PTPN11, NRAS, KRAS, CBL*, or *NF1*. The first three subtypes (*PTPN11, NRAS, KRAS*) are characterized by heterozygous somatic gain-of-function mutations in non-syndromic children, while JMML in neurofibromatosis type 1 and JMML in children with CBL-syndrome are characterized by germ line RAS disease and acquired biallelic inactivation of the *NF1* or *CBL* gene in hematopoietic cells (12, 37). Clinical presentation as well as outcome differs between these 5 JMML subtypes. Secondary genetic alterations like *ASXL1, EZH2, SETBP1, JAK3*, and mutations in spliceosomal genes often result in disease progression. Generally, a wide variation exists regarding the clinical course of the disease. In about 15% of children, most frequently in CBL mutated disease, spontaneous regression occurs. The majority of children affected by JMML, however, require allogeneic transplantation to cure the disease. An allogeneic stem cell transplantation from a histo-compatible sibling or HLA-matched unrelated donor results in a disease-free survival of 52% in a study from 2005 by Locatelli et al. (62). In an earlier study (1997) the probability of survival at 10 years was 0.39 for children having received allogeneic stem cell transplantation and 0.06 for children that did not receive HSCT (37). Variables like age, level of HbF, platelet count, or, more recently described, genome-wide DNA methylation profiles may be helpful to predict the clinical course.

aCML is a rare, *BCR-ABL1*-negative, MDS/MPN overlap syndrome characterized by leukocytosis, granulocytic dysplasia, and a dismal prognosis. It was first described as a variant CML lacking the Philadelphia chromosome, but diagnostic criteria have evolved since. However, it can still be challenging to distinguish aCML from other MPNs like chronic neutrophilic leukemia or from other MDS/MPN like MDS/MPN-U, as the diagnosis largely relies on morphologic criteria. Its frequency is not well known, but it is estimated to account for 1-2% of *BCR-ABL1*-positive CML (~0.5-2/100000) (44, 70–73). The disorder affects elderly patients with a median age of 62-73 years and a male predominance (15, 32, 43–47, 97, 98). The clinical picture is comparable to Philadelphia positive CML including elevated WBC with co-occurrence of mature and immature cells of the granulocytic lineage, splenomegaly, and mild to moderate anemia. Typical for aCML, however, are severe dysplastic features predominantly in the granulocytic lineage. Also, in contrast to classical CML, the genetic basis of the disease is heterogeneous, with *SETBP1* and *ETNK1* mutations being recurrent, but several other mutations, typical for MDS and/or MPNs can be found as well (15). Cytogenetic abnormalities are less frequent and occur in 20-40% of patients only (43, 44, 46). Median survival varies between 10 and 25 months (43, 44, 46, 47). AML evolution occurs in about 31-40% of patients with a median time to AML of 11-18 months (43, 44, 46).

Of the MDS/MPN overlap syndromes, MDS/MPN-U is the least well defined. It encompasses such patients, that show features of myelodysplastic as well as myeloproliferative disease, who do not fit into one of the other 4 subgroups. The diagnosis is extremely rare, accounting for less than 2% of MDS (49). In the Duesseldorf MDS registry it currently accounts for only 0.05% of all MDS (35). Patients with MDS/MPN-U are relatively old with a median age of 70 years and show the male predominance that is seen in other MDS/MPN subgroups. Regarding cytogenetic features, the percentage of abnormal, including complex karyotypes is higher when compared to other MDS/MPN. Of the molecular abnormalities found in patients with MDS/MPN-U the *JAK2-V617F* mutation is relatively frequent in contrast to the other overlap syndromes except MDS-RS-T. Clinical characteristics are not well established and often seem to show similarities with one of the other MDS/MPN-subgroups. Thus, MDS/MPN-U is rather a mixture of patients not fulfilling all criteria for the diagnosis of one of the other MDS/MPN subtypes (i.e. not enough peripheral monocytes to fulfill the diagnosis CMML or slightly less than 450.000 thrombocytes not fulfilling the criteria for MDS-RS-T, WBC too low for aCML,…). This is, of course, due to the fact, that all thresholds are more or less arbitrary. This fact might be unsatisfactory, but as thresholds are necessary, a solution might be to form subgroups of “CMML-like MDS/MPN-U” and “MDS-RS-T like MDS/MPN-U” and so on or to allow the diagnosis of pre-CMML syndromes like oligo-monocytic CMML (86, 87). Patients should be checked at regular intervals whether they still fit into the MDS/MPN-U category or might be transferred into a better defined MDS/MPN subtype. With a median survival of 1-2 years, survival of patients with MDS/MPN-U is generally poor. However, some subgroups like the “MDS-RS-T like MDS/MPN-U” might do better than others.

Refractory anemia with ringed sideroblasts associated with marked thrombocytosis (MDS-RS-T) is the latest “member” in the group of MDS/MPN. It was proposed as a provisional entity in the WHO 2001 classification of myeloid neoplasms. The latest WHO-classification has now recognized MDS/MPN-RS-T as a formal subgroup of the MDS/MPN overlap syndromes. MDS/MPN-RS-T shares clinical features with MDS-RS-SLD and essential thrombocythemia. It is characterized by the co-occurrence of ringed sideroblasts in the bone marrow (≥15%), together with an increased platelet count (≥450 000/µl) and large, atypical megakaryocytes. As for most of the other MDS/MPNs epidemiological data is scarce, due to the rarity of the disease. Its frequency can be estimated to be below or about 1% of all MDS (49, 50, 69). In our current MDS registry it accounts for 1,45% of all MDS (35). The median age at presentation ranges from 71-75 years (13, 39–42). In contrast to other MDS/MPN the male predominance seems less pronounced but varies between studies (13, 39–42). Hepato-splenomegaly and extramedullary involvement appear to be less frequent compared to other MDS/MPD, same as the “MPD-typical” constitutional symptoms, although data on these clinical features is still very limited. In addition, the prognosis is generally better than that of other MDS/MPD as it resembles the two relatively “benign” diseases MDS-RS-SLD on the one hand and ET on the other hand, leading to a relatively low risk of leukemic transformation, but also to an increased risk of thromboembolic events and an often symptomatic anemia as the typical presentation of this unique MDS/MPD subgroup. Regarding cytogenetics, about 80% of patients exhibit a normal karyotype. Gene mutations, conversely, are frequent and observed in >90% of patients (13, 14). The most frequent are *SF3B1* as well as *JAK-2* mutations. Patients with RARS-T have a shorter overall (76 *vs*. 117 months) and leukemia-free survival than patients with essential thrombocythemia along with a comparable risk of thromboembolic complications (3.6 *vs*. 3.9/100 patient years). On the other hand, they exhibit a longer survival (76 *vs*. 63 months), but a higher risk of thrombosis when compared to patients with MDS-RS (3.6 *vs*. 0.9/100 patient years) (39).

Two groups that are not recognized as (separate) entities within the MDS/MPN but show unique features and an overlap of both MDS and MPN are patients with del(5q) and *JAK2 V617F* mutation and patients with isolated isochromosome (17q). These groups are small, but usually show the typical overlapping symptoms of both myelodysplastic and myeloproliferative disease. MDS with del(5q) and *JAK2* are currently subsumed under MDS with isolated del(5q). This makes sense on the one hand since patients appear to have a comparable prognosis when compared to patients with isolated del(5q) without the *JAK2* mutation and treatment with lenalidomide appears to be active in both subgroups. However, lenalidomide shows activity in MPNs like myelofibrosis as well. On the other hand, the most recent and most extensive publication on this small subgroup of MDS patients by Sangiorgio et al. (21) shows that median cell counts regarding platelets, but also WBC, and even red blood cell counts, are higher when compared to MDS with del(5q) and *JAK2*-wildtype. 3 patients did not even meet the criteria for MDS and del(5q) because they lacked sufficient cytopenias. In addition, all 3 patients with data available showed splenomegaly, 4 of 5 patients with available bone marrow histology were hypercellular and all these patients had grade 1 or 2 fibrosis. Dysplasia in the erythroid or granulocytic lineage was lacking. Still, megakaryocytes were not typical for MPN, but clearly exhibited dysplastic, del(5q) like features, with hypo- and monolobulated nuclei, while large hypernucleated forms existed as well. Thus, one could argue that such patients, according to their clinical presentation, might better be recognized as MDS/MPN overlap syndromes than MDS. In this study 12.7% of all MDS with isolated del(5q) were found to have a *JAK2 V617F* mutation. Others found a slightly lower frequency (22). The *JAK2 V617F* mutation identifies a subgroup of MDS patients with isolated deletion 5q and a proliferative bone marrow.

Isolated isochromosome (17q) can be a finding within complex karyotypes occurring in different myeloid malignancies, but also, rarely, exists as sole chromosomal abnormality. In this case, it often presents as MDS/MPN overlap syndrome. The median age is around 60 years, with the typical male predominance (23–30). Patients often present with anemia, leukocytosis, and splenomegaly. The bone marrow is hypercellular, often exhibiting some grade of fibrosis, and dysgranulopoiesis, including hypo- and non-segmented forms, ring nuclei, hypogranularity, and chromatin clumping is typically prominent. The blast count is usually low. Monocytosis occurs frequently, thus many cases are currently subsumed under CMML. A few might present like aCML, but most other cases can only be placed in MDS/MPN-U. As patients with this unique cytogenetic feature share many clinical features and seem to have a relatively uniform poor prognosis and high risk of leukemic evolution it is a matter of discussion, whether it might make sense to form a new, cytogenetically well-defined subgroup of MDS/MPN. However, in the Duesseldorf MDS registry currently only one patient with isolated isochromosome (17q) can be detected. This patient was diagnosed as CMML.

## Conclusion

The WHO-category MDS/MPN encompasses a unique group of clonal myeloid neoplasms exhibiting hybrid features of myelodysplastic as well as myeloproliferative malignancies. As most entities are quite rare, epidemiological data is sparse. In adults the most frequent MDS/MPN by far is CMML, followed by MDS/MPN-RS-T. aCML and MDS/MPN-U are extremely rare diseases and not very well defined. An appropriate diagnosis and classification are difficult, but essential for further prognostication and treatment decisions. Although diagnosis of most subtypes is still largely based on morphologic criteria, diagnosing MDS/MPD properly should require a comprehensive clinical and laboratory assessment with thorough integration of morphological, immunophenotypic, genetic, as well as clinical examination. While single gene mutations might occur in different MDS/MPN or other myeloid diseases certain gene combinations may be more specific for certain subtypes and might aid in determining the correct diagnosis (69). Despite an enormous gain of knowledge regarding molecular genetics and in some subgroups pathophysiology as well we are still far from satisfactory treatment options in this rare and heterogeneous group of myeloid overlap syndromes.

## Author Contributions

AKu, UG, and AKa contributed to conception and design of the paper. AKu wrote the first draft of the manuscript. All authors contributed to manuscript revision, read, and approved the submitted version.

## Conflict of Interest

The authors declare that the research was conducted in the absence of any commercial or financial relationships that could be construed as a potential conflict of interest.

## Publisher’s Note

All claims expressed in this article are solely those of the authors and do not necessarily represent those of their affiliated organizations, or those of the publisher, the editors and the reviewers. Any product that may be evaluated in this article, or claim that may be made by its manufacturer, is not guaranteed or endorsed by the publisher.
